# ccf-mtDNA as a Potential Link Between the Brain and Immune System in Neuro-Immunological Disorders

**DOI:** 10.3389/fimmu.2019.01064

**Published:** 2019-05-09

**Authors:** Stefano Gambardella, Fiona Limanaqi, Rosangela Ferese, Francesca Biagioni, Rosa Campopiano, Diego Centonze, Francesco Fornai

**Affiliations:** ^1^I.R.C.C.S Neuromed, Via Atinense, Pozzilli, Italy; ^2^Human Anatomy, Department of Translational Research and New Technologies in Medicine and Surgery, University of Pisa, Pisa, Italy; ^3^Multiple Sclerosis Clinical and Research Unit, Department of Systems Medicine, Tor Vergata University, Rome, Italy

**Keywords:** mitochondria, mtDNA, circulating cell-free mtDNA, exosomes, neuro-immunity

## Abstract

Fragments of mitochondrial DNA (mtDNA) are released outside the cell and they appear to persist in extracellular fluids as circulating, cell-free, mtDNA (ccf-mtDNA). When compared to nuclear DNA, such a double stranded mtDNA is more resistant to nuclease degradation. In fact, it is stable extracellularly where it can be detected in both plasma and cerebrospinal fluid (CSF), here acting as a potential biomarker in various disorders. In neurological diseases (Alzheimer's disease, Parkinson's disease and end-stage progressive Multiple Sclerosis), a decreased amount of CSF ccf-mtDNA is related with progressive cell dysfunction. This suggests an alteration in neuronal mtDNA levels (mtDNA replication, degradation and depletion) in vulnerable brain regions at early stages of neurodegeneration leading to reduced mtDNA release, which takes place before actual cell death occurs. On the other hand, elevated CSF ccf-mtDNA levels are reported in acute phases of relapsing-remitting Multiple Sclerosis (RRMS). This occurs during acute inflammation, which anticipates the neurodegenerative process. Thus, an increase in inflammatory cells in the affected regions is expected to add on mtDNA release into the CSF. In addition, similarly to bacterial DNA, the non-methylated CpG sites of mtDNA, which activate innate immunity and inflammation, are likely to participate in the molecular mechanisms of disease. Thus, ccf-mtDNA may represent a powerful biomarker for disease screening and prognosis at early stage, although its biological role may extend to generating the neurobiology of disease. The present manuscript discusses recent experimental findings in relationship with clinical evidence comparing neuro-immunological features of neurodegenerative disorders with frankly neuro-infectious diseases.

## Introduction

Within eukaryotic cells, mitochondria play an important role in energy metabolism, generation of free radicals, calcium homeostasis, and apoptosis (1). Each cell may contain up to thousands of mitochondria being each mitochondrion gifted with its own genome (mtDNA), which replicates independently of nuclear DNA (2). In humans, mtDNA is significantly smaller when compared with nuclear DNA (16.569bp vs. 3.2 billion bp), and it possesses only 37 genes, among which 13 encode proteins belonging to the respiratory electron transport chain (3). Unlike nuclear DNA, mtDNA is devoid of protective histones and sophisticated DNA repair mechanisms, which makes it vulnerable to genotoxic stimuli including oxidative stress (4, 5). In fact, high levels of reactive oxygen species (ROS) are generated around mtDNA during oxidative phosphorylation occurring in mitochondria. Such an oxidative environment contributes to a high susceptibility of mtDNA to mutagenesis. In fact, mtDNA possesses roughly a 10- to 200-fold higher rate of mutagenesis than nuclear DNA under a comparable oxidative stress environment. This may be detrimental for those high-energy-demanding and post-mitotic cells including neurons and myocytes, which are mostly sensitive to altered respiratory chain activity and ROS-mediated damage yielded by mtDNA changes (6). Such a specific vulnerability of mtDNA determines the occurrence of a detectable amount of mitochondrial DNA fragments, which are released into the bloodstream as circulating, cell-free fragments (ccf-mtDNA). These correspond to double-stranded DNA molecules, which are biologically fragmented into both short (lower than 1 Kb) and long (up to 21 kb) segments (7). Due to a striking structural similarity which is evolutionary derived from bacterial DNA, mtDNA fragments activate innate immunity and inflammation through a common molecular pathway operating in a number of etiologically different disorders (4, 8–12). In this way, ccf-mtDNA generate key, yet double-faceted inflammatory responses including antimicrobial immunity and neuro-immunological disorders. As for most molecules involved in the immune response, the bulk of evidence concerning the measurement of ccf-mtDNA and its role in physiology and disease stems from studies carried out outside the CNS. In fact, ccf-mtDNA has been analyzed in various clinical conditions like neoplasia, trauma, infections, stroke and cardiovascular diseases, where it has been tested as diagnostic and predictive biomarker. Only recently, mtDNA started being evaluated in neurological disorders of both sterile and infectious origin. In line with the higher resistance of mtDNA to nuclease-dependent degradation compared with nDNA (13), mtDNA persists as ccf-mtDNA within extracellular fluids including the CSF. Thus, ccf-mtDNA may be a potential biomarker of cell death and non-specific tissue injury; if further validated, in the near future this is supposed to become an innovative diagnostic tool in early stage screening and prognosis of several disorders (7). Nonetheless, the biological role of ccf-mtDNA and its fragments is still controversial and it needs to be fully understood. Therefore, in the present manuscript we discuss the role of ccf-mtDNA as a potential link between the brain and immune system, which may provide novel insights into neurological disorders.

## ccf-mtDNA and Neuro-Infections

**Figure 1:**
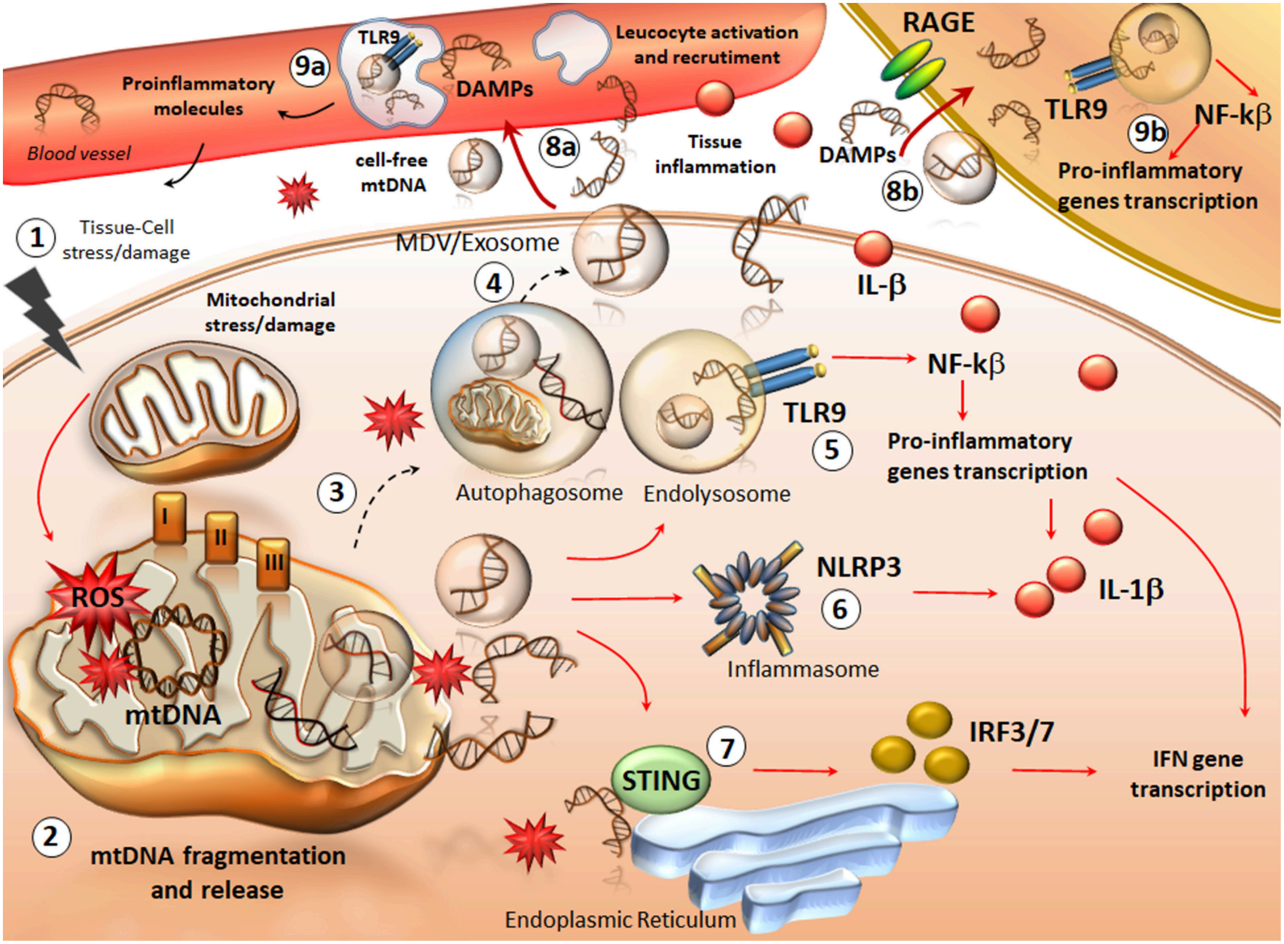
An overview of mtDNA fragments life: from their generation to spreading and their role as DAMPs in inflammation. Following cellular stress/injury (1) mitochondria are compromised due to either a direct damage or an increased attempt to cope with metabolic cell demand. Thus, an increased amount of ROS in the three complexes of the respiratory chain, directly affects mtDNA integrity (2). This leads to the accumulation of oxidized mtDNA fragments, which along with ROS, are released in the cytosol as free molecules or engulfed into mitochondrial derived vesicles (MDV). The accumulation of mtDNA within a highly oxidative environment is likely to overcome the attempt of autophagy to digest damaged mitochondria and mtDNA fragments (3). This leads to the spreading of free- or exosomes/MDV engulfed- mtDNA fragments in the extracellular space (4). At the same time, intracellular mtDNA fragments trigger an inflammatory response already within the cytosol. In fact, mtDNA behave as DAMPs to activate TLR9 on the endo-lysosomal membrane (5). This promotes the activation of NFkB and the transcription of pro-inflammatory genes such as interferons (INFs) and pro-interleukines (ILs). Such an effect is magnified by an mtDNA-depended activation of two other components (6, 7), the nucleotide-binding oligomerization domain (NOD)-like receptor family pyrin domain containing-3 (NLRP3) (inflammasome) and the stimulator of interferon genes (STING) system. In this way, cytokines including IFN3/7 and IL-1β are produced and released extracellularly along with cell-free mtDNA. ccf-mtDNAs are spread into the bloodstream where they add on IL-1β activity to recruit and activate leucocytes while promoting tissue leukocyte infiltration (8a). Within blood leucocytes, ccf-mtDNA behave as DAMPs to promote inflammatory reaction, which favors cytokines release from the blood (9a). Again, ccf-mtDNA are spread within neighboring cells (such as tissue resident macrophages), either via exosome endocytosis or via binding to receptors for advanced glycated end-products (RAGEs) (8b). This is key to activate macrophages and fuel mtDNA-TLR9 dependent inflammation (9b). The result of such a domino-like cascade promoted by ccf-mtDNA is a vicious inflammatory cycle, which fosters tissue and cell injury along with progressive mitochondrial damage (1).

Mitochondria are evolutionarily derived from energy-producing alpha-bacteria, engulfed by archezoan cells approximately 2 billion years ago leading to a symbiotic relationship that built up eukaryotic cells (14). Mitochondria share several features with bacteria, including the double-membrane structure and a circular genome which replicates independently of nuclear DNA and possesses non-methylated CpG sites. In the light of such a similarity, once released in either cytosol or extracellular space, mtDNA fragments behave as danger-associated molecular patterns (DAMPs) to activate innate immunity and inflammation just like pathogen-associated molecular patterns (PAMPs). This occurs through a molecular cascade which consists of binding to Toll-like receptor 9 (TLR9) and subsequent activation of the stimulator of interferon genes (STING) pathway (9–11, 15). (Figure 1). Therefore, DAMPs accumulation activates resident macrophages and fosters tissue infiltration by leukocytes (16, 17). Such an overlapping molecular mechanism between DAMPs and PAMPs may also lead to indistinguishable clinical responses following infective and non-infective insults, as exemplified by neurological and even systemic inflammatory syndromes (18–20). In this context, while the detection of pathogen-associated genome remains seminal for the clinical diagnosis, quantification of ccf-mtDNA is emerging as a biomarker of tissue damage and/or disease severity induced by pathogens. For instance, increased levels of ccf-mtDNA and iron biomarkers are detected in CSF and plasma from untreated HIV-infected patients, which associates with HIV viral load and mild neurocognitive impairment (20–22). This suggests that active viral replication perturbs mitochondrial integrity leading to a progressive extrusion of mtDNA fragments. Contrariwise, meningitis-related pathogens (Streptococcus pneumonia and Neisseria meningitis) produce a dramatic mitochondrial depletion and dysfunction, which associates with decreased plasma mtDNA levels and a less efficient antimicrobial immune response assessed in animal models (23–25). Likewise, a quite selective mitochondrial damage and reduction in mtDNA levels is detected in autopsy brains from Herpes simplex (HSV-1) encephalitis patients (26), though studies quantifying ccf-mtDNA in this specific case are still missing so far. Nonetheless, these findings suggest that a correlation between ccf-mtDNA levels and disease severity stems from the degree of mitochondrial impairment and mtDNA damage. It is likely that in the early phase of infection, when mitochondrial and plasma membrane damage is slight, the spread of ccf-mtDNA serves as an attempt to activate antimicrobial immunity (27) and to guarantee the preservation of mitochondrial function by removing dysfunctional mtDNA fragments. In fact, even when the plasma membrane is intact, fragments of mutated mtDNA are compartmentalized within cytosolic organelles such as exosomes and then released extracellularly along with other constituents such as micro RNA (miRNA) (28). This latter mechanism would guarantee the preservation of mitochondrial function by removing dysfunctional mutated DNA fragments (29–31). This is supported by recent work from *C. elegans* neurons, which expel dysfunctional mitochondria when exposed to neurotoxic stress (31). On the other hand, persistent or severe insults of either oxidative or infective nature can damage cell integrity while producing apoptosis or necrosis, which is considered as the main mechanism leading to mtDNA extrusion from the cell or release into the blood (32, 33). Once released extracellularly, mtDNA fragments may also act as toxic molecules that further impair mitochondrial function, cell integrity and tissue repair (34). It is remarkable that the role of ccf-mtDNA as a potential biomarker extends beyond infectious disorders to encompass neurodegenerative and neuro-immune disorders, where the double-faceted mechanisms of ccf-mtDNA release are very much recapitulated.

## ccf-mtDNA and Neurodegeneration

Recent studies linked the amount of CFS ccf-mtDNA to neurodegeneration in patients affected by Alzheimer's disease (AD) and Parkinson's disease (PD). In detail, a low concentration of ccf-mtDNA was reported in the CSF of asymptomatic subjects with familial and sporadic AD, suggesting that an alteration in mtDNA replication or degradation precedes biomarkers of neuronal damage in AD (30). In fact, a reduction in the amount of mtDNA within pyramidal neurons in AD hippocampi parallels the reduction of ccf-mtDNA levels in early-stages of AD. Unfortunately, other studies failed to replicate these data on a large series of well-characterized cohorts of AD patients (35). Nonetheless, a significant reduction of CSF ccf-mtDNA was recently reported in sporadic PD patients, suggesting that reduced CSF ccf-mtDNA may be a biomarker for early stages of neurodegeneration (36). The specific role of reduced ccf-mtDNA remains unknown (37, 38). The occurrence of reduced amount of ccf-mtDNA in neurodegenerative disorders may appear as a contradiction when considering the occurrence of cell death, which is expected to massively release mtDNA. This would elevate rather than depress ccf-mtDNA levels. This contradiction is even stronger when considering that apoptotic cell death, which occurs in neurodegeneration, should further elevate the occurrence of mtDNA levels within dying neurons. Therefore, it is unexpected that in PD and AD low ccf-mtDNA levels are detected. Such a contradiction turns out to be only apparent since mitochondria, as well as neuronal mtDNA levels, are reduced during early stages of cell pathology. Thus, it is likely that a suppression of baseline release of mtDNA occurs following mitochondrial loss. This would explain why low ccf-mtDNA levels, which may anticipate the occurrence of neuronal death, are detected at early stages of neurodegeneration. This is supported by studies showing that reduced neuronal mtDNA copy number in neurodegenerative disorders is related with a reduction in cell energy (38, 39). Remarkably, tissue-specific depletion of mtDNA occurs in PD and AD patients compared with age matched controls (40, 41). In detail, in dopaminergic (DA) substantia nigra neurons of healthy individuals, mtDNA copy number increases with age in order to maintain the pool of wild-type mtDNA population in spite of accumulating deletions (42). Such an upregulation fails to occur in PD patients, resulting in depletion of wild-type mtDNA (42) and an increase in mtDNA deletions (40, 43). This very same mechanism characterizes hippocampal pyramidal neurons in AD patients, where a reduction in mtDNA levels goes along with an increase in mtDNA deletions (41, 44). Studies quantifying the amount of ccf-DNA in neurodegenerative disorders are still limited, thus the clinical significance remains to be elucidated. Likewise, experimental studies are needed to disclose the molecular mechanisms bridging ccf-mtDNA with neurodegeneration. Experimental studies suggest that a dysfunction of Parkin and PINK1-related mitophagy may be key in contributing to mitochondrial decline and accumulation of mtDNA mutations in DA neurons of sporadic PD (45). This was assessed in mice homozygous for a proofreading deficiency in DNA polymerase gamma (PLOG), which leads to mitochondrial dysfunctions and progressive accumulation of mtDNA mutations/deletions. As recently demonstrated, PLOG precludes the formation of mtDNA deletions by degrading linear mtDNA fragments (46), which would otherwise be released as ccf-mtDNA. Several patients with *POLG* mutations and multiple mtDNA deletions also present with early onset parkinsonism (47). However, Pickrell et al. (45) demonstrated that a frank loss of DA neurons recapitulating PD in POLG mutated mice, occurs only following Parkin deletion. This suggests that Parkin-mediated mitophagy compensates for mtDNA mutations by rescuing mitochondrial functions to grant DA neuron survival. The role of mitophagy in mitochondrial turnover and ccf-mtDNA release appears critical also in LRRK2-related PD. In fact, ccf-mtDNA emerged as a potential pathophysiological marker in PD patients with p.G2019S mutation in LRRK2 (48), which affects mitochondrial function/dynamics by slowing down mitophagy (49–51). Variant p.G2019S has a reduced age-dependent penetrance (52), but the mechanisms whereby a significant percentage of asymptomatic LRRK2 mutation carriers never develop PD remain unknown. Intriguingly, Podlesniy et al. (48) showed that p.G2019S carriers with PD exhibit a higher concentration of mtDNA in CSF, compared with asymptomatic carriers and with idiopathic PD patients. These findings suggest that different biochemical, and inflammatory pathways may underlie the significance of ccf-mtDNA as a biomarker of neurodegeneration in different forms of PD. Studies on other neurodegenerative disorders suggest that the disease stage plays a seminal role. In fact, ccf-mtDNA has been analyzed in multiple sclerosis (MS), a neurodegenerative disorder possessing a strong inflammatory response. When ccf-mtDNA were analyzed in ventricular CSF (vCSF) at end-stage of progressive multiple sclerosis (PMS), when neurodegeneration is pronounced, ccf-mtDNA levels were depressed. This is consistent with other neurodegenerative disorders such as AD and PD (29, 30). In line with this, when compared with other markers for neurodegenerative disease, the reduction in ccf-mtDNA occurs concomitantly with an increase in the levels of glial fibrillary acidic protein and S100 calcium binding protein B (53).

## ccf-mtDNA Levels are Elevated in Neurological Diseases When Strong Inflammation is Present

The role of mtDNA is emerging in immune-mediated inflammatory diseases outside the CNS, such as rheumatoid arthritis. In this chronic relapsing disorder, autoimmune response mostly affects the joints and mtDNA has been detected both in plasma and synovial fluid (54). Intra-articular injection of oxidized mtDNA, but not nuclear DNA, triggers inflammatory arthritis in mice (55). There are now numerous studies using *in vivo* injection of mtDNA to provoke local and/or systemic inflammation (8, 56–58). This is not surprising since mtDNA contributes to inflammation at multiple levels when tissue or cell homeostasis is perturbed. In fact, damaged mtDNA released into the cytosol activate the inflammasome and NF-κB by binding to TLR9 (9, 10, 12, 59). Moreover, mtDNA, which is released in the cytosol due to mitochondrial stress, activates the STING pathway and fosters the production of cytokines (4, 11, 60). Noteworthy, this occurs along with pentraxin-3 release (61), which is a strong modulator of the neuro-immune response (62, 63). Remarkably, in contrast to neurodegeneration, which associates with a reduction of CSF ccf-mtDNA, CNS disorders featuring a strong inflammatory response are characterized by elevated plasma mtDNA levels. In fact, elevated CSF ccf-mtDNA occur in relapsing-remitting MS (RRMS) (64, 65), which possess an acute inflammatory response (66). Thus, it is likely that the increase in ccf-mtDNA observed in RRMS is a direct consequence of increased activation of inflammatory cells, which release mtDNA into the CSF (67). A persistent inflammatory reaction may recruit circulating immune cells, while triggering a systemic response through the activation of mtDNA-induced inflammatory pathways. In this way, a vicious circle occurs where inflammatory cytokines and ROS may induce further damage to mitochondria and mtDNA. In this scenario, elevated ccf-mtDNA concentration in MS may reflect early, active inflammatory activity, which eventually culminates in mitochondrial damage, neural loss and brain atrophy (65). In this condition, the measurement of ccf-mtDNA concentration configures as a potential biomarker for acute inflammatory stress. Whether this phenomenon is specific for MS or it rather reflects generic neuro-inflammation, still needs to be investigated (64). Since mitochondrial damage occurs in active MS lesions (68), one could speculate that mtDNA in the CSF may act as a DAMP. Considering mtDNA as a DAMP in MS, may explain the “inside-out theory” which suggests that inflammation is secondary to a primary intrinsic process within neurons or other cells such as oligodendrocytes (69). This neuro-immune concept consists in the formation of intracellular compounds, which trigger biochemical cascades leading to immunity activation (inflammasome) which once released from the cell recruit in turn a focal immune response. In this scenario, the “inside” mtDNA fragment would be the inflammatory stimulus, which clusters the intracellular cascade leading to a molecular complex, which triggers the immune response. Once such a complex is exposed “out” of the cell, immunity is strongly activated. This is supported by studies showing that ccf-mtDNA may be useful as a biomarker to predict treatment outcomes in MS. In fact, compared with untreated patients, decreased levels of mtDNA occur in patients receiving fingolimod, which limits autoreactive inflammation in the CNS by acting on sphingosine-1-phosphate (S1P) receptors, which are present on peripheral immune- as well as glial and nerve cells (65). This warrants for further studies focusing on disease modulatory treatments in order to understand how they affect ccf-mtDNA levels, inflammation and CNS cell injury.

## The Crosstalk Between miRNAs and Mitochondria in Neuro-Inflammation

**Figure 2:**
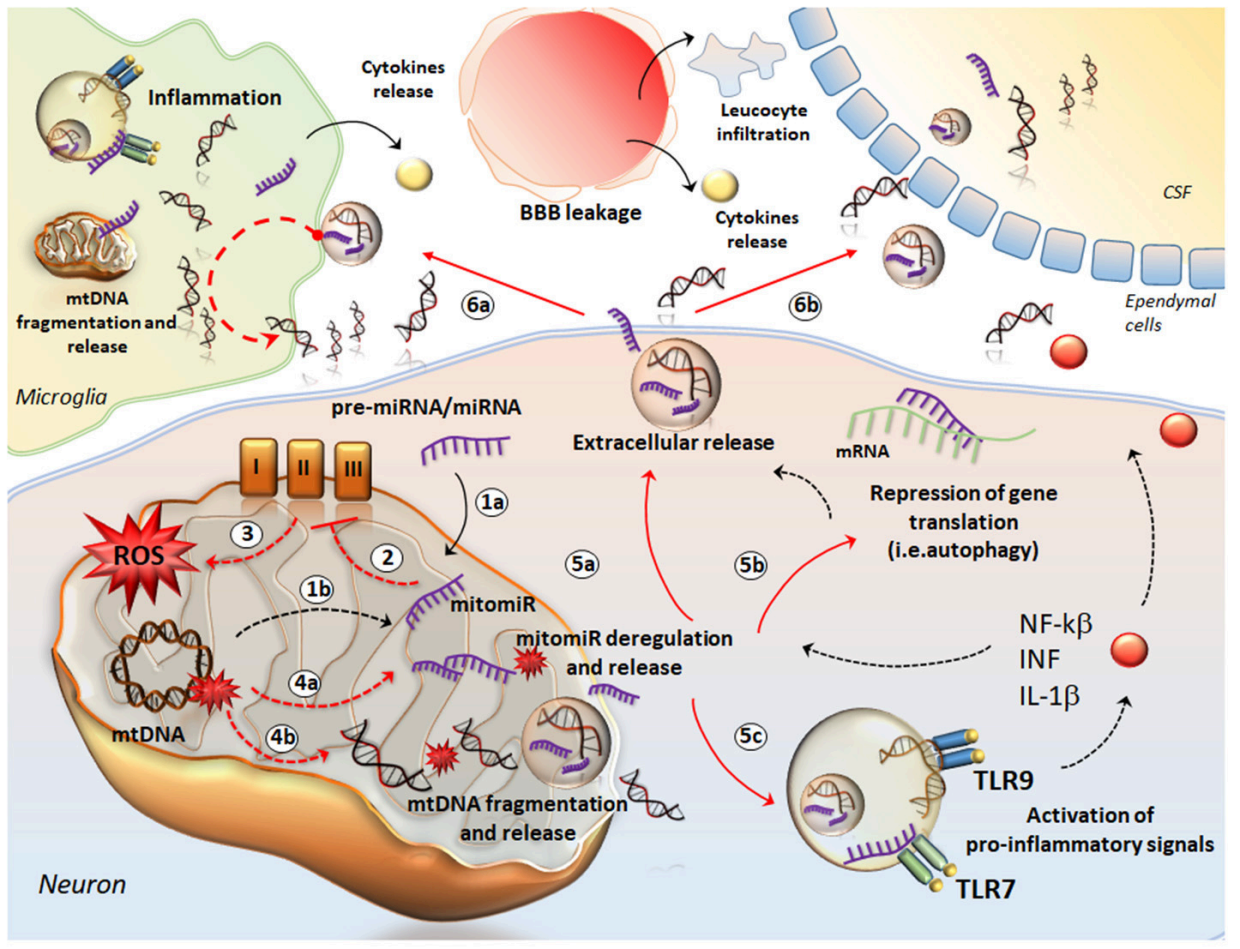
The crosstalk between mtDNA and miRNA in neuro-inflammation. The cartoon provides a schematic description of how mtDNA and mitomiR may behave as a single entity to promote neuro-inflammation. mitomiRs may derive either from cytosolic miRNA/pre-miRNA exported and processed within mitochondria (1a) or directly from mtDNA (1b). mitomiRs disrupt the respiratory chain complexes (2) and increase ROS production (3) thus producing mitochondrial damage, mitomiR dysregulation (4a), mtDNA fragmentation (4b) and spreading in the cytosol and further, in the extracellular space (5a). In the cytosol, mitomiRs repress a wide array of genes, among which those encoding for mitophagy (5b), thus exacerbating mitochondrial damage and cell stress. This enhances the extracellular release of mitomiRs and mtDNA (5a). In addition, both mitomiR and mtDNA behave as DAMPs to initiate an inflammatory response already within neurons and promote the release of pro-inflammatory cytokines (5c). In the extracellular space, mitomiRs and ccf-mtDNA spread into microglia (6a) to activate inflammation and again, to promote mitochondrial damage. This translates into fragmentation of mtDNA within glial cells and further release of cff-mtDNA and mitomiRs. Thus, these fragments along with pro-inflammatory molecules exacerbate the inflammatory response at the level of tissue stroma, which is magnified by the massive leucocyte infiltration and cytokines release fostered by the leakage of blood brain barrier (BBB). At the same time, ccf-mtDNA and mitomiRs cross the ependymal wall to reach the CSF (6b) and then the blood, where they are detectable as biomarkers of neuro-inflammation.

The foregoing discussion is centered on the potential role of ccf-mtDNA as an emerging biomarker of neurological disorders. However, it is mandatory to relate the neuro-inflammatory significance of ccf-mtDNA to other, well-known circulating fragments of nucleic acids, namely micro RNAs (miRNAs). Briefly, miRNAs are single-stranded, non-coding RNA fragments, which behave as post-transcriptional gene modulators by inhibiting messenger RNA (mRNA) translation or promoting their degradation. Remarkably, recent studies demonstrated that besides nuclear and cytosolic compartments, miRNAs are found within mitochondria (70, 71). These latter are named mitomiRs and include miR-155, miR-181c, miR142-3p/5p and miR146a. It is remarkable that, mitomiRs possess a double origin: (i) they are nuclear genome-derived cytosolic miRNA precursors, exported and processed within mitochondria, and/or (ii) they originate directly from mtDNA (72). This issue integrates mtDNA and miRNA in single entities, which may be dysregulated in a variety of CNS disorders including AD, PD and MS. Oxidative stress, which is tightly bound to mitochondrial function and integrity, is one of the leading causes of RNA oxidation and miRNA pathway dysregulation (73, 74). This suggests that, miRNA dysregulations may derive from mitochondrial damage (Figure 2). Alternatively, miRNA dysregulations occurring in the nucleus or cytosol may translate into mitochondrial and mtDNA alterations once these miRNA translocate within mitochondria. In any case, miRNA dysregulations produce mitochondrial dysfunction via repression of local mtDNA transcription (75). For instance, miR-13a and miR-181c impair cytochrome oxidase complex I and III, leading to a massive increase in ROS, which may lead to mtDNA fragmentation. This is key for spreading mtDNA fragments from mitochondria within and outside of the cell, and for releasing mitomiRs as well. In fact, mitochondria are the main vehicles, which deliver miRNAs toward intracellular compartments to modulate expression of a broad array of genes (76). Such an abnormal spreading of miRNAs within the cytosol suppresses mitophagy (71, 77), thus impeding the removal of damaged mitochondria, mtDNA and miRNAs. An imbalance between mitochondrial clearance and biogenesis occurs in several neurological disorders (78–80). In line with this, just like ccf-mtDNA, degradation-resistant miRNAs circulate in extracellular fluids within exosomes [Figure 2, (81, 82)]. Again, circulating miRNAs such as miR-155, miR 146a and let-7 are implicated in neuro-inflammation by acting as DAMPs to activate TLR7 (83–86) in a way, which is reminiscent of ccf-mtDNA. This appears not to be coincidental when considering that, these DAMP-like miRNAs reside in mitochondria and their activity is elicited by NF-κB, (which in turn is induced by mtDNA fragments). These findings suggest that the commonalities between mtDNA and miRNAs as potential biomarkers for neuro-inflammatory disorders are tightly bound to their mitochondrial nature. A dysregulation of miRNAs, mitomiRs, and mtDNA perturbs mitochondria and affects the immune response in the brain. In turn, mitochondrial damage starts the spreading of membrane-free or exosomal constituents to promote inflammation at distant sites Figure 2. At first glance, it appears that a broad range of effects produced by miRNAs may not allow for a direct correlation with neuro-inflammation when compared with a quite specific role of ccf-mtDNA. Nonetheless, the interaction between miRNA, mitochondria and circulating mtDNA fragments should be statistically integrated to comprehend their role as biomarkers of neuro-inflammation.

## Conclusions

These findings cast the hypothesis that variations in mtDNA levels within the CSF may not simply and passively reflect neuronal integrity, since an active role of mtDNA in the neurobiology of disease may underlie a specific cascade of neuro-inflammatory events. This appears to be quite specific for those neurological disorders, which are triggered by inappropriate immune responses. An active role of extracellular mtDNA is more intriguing than a mere index of neuronal integrity. In fact, there are neurological conditions where a frank neuronal loss occurs without variations in mtDNA levels. This is the case of Creutzfeldt-Jakob disease (CJD), in which massive neuronal loss occurs despite being mtDNA levels comparable with controls. Thus, it is likely that the specific molecular mechanisms underlying early stage of neuronal degeneration are key in conditioning mtDNA levels. For instance, the primary involvement of mitochondrial damage producing early mitochondrial loss may explain why mtDNA levels are reduced in neurodegenerative conditions such as PD and AD, while the absence of an early mitochondrial pathology in CJD would explain the occurrence of normal mtDNA levels. The increase in mtDNA would require an early, strong involvement of neuro-immunological mechanisms which does not occur neither in AD/PD (48, 64) nor in CJD but is prominent in MS. In fact, mtDNA in MS could be actively released in response to a stimulus, similar to the release by eosinophils in response to bacterial infection (27). In this scenario, ccf-mtDNA could be a primary intrinsic process of neuropathology. Nonetheless, the role of ccf-mtDNA represents a novel and emerging research field in the context of neurological disorders, and as such, it remains rather preliminary and speculative. This calls for further studies aimed at elucidating whether and how ccf-mtDNA contributes to and reflects disease onset and progression.

## Author Contributions

SG coordinator of the sections about ccf-mtDNA, participate in drafting. FL pubmed research and state of the art about ccf-mtDNA, production of figures. RF pubmed research and state of the art about ccf-mtDNA and neurodegeneration. FB pubmed research and state of the art about ccf-mtDNA levels are elevated in neurological diseases when strong inflammation is present. RC pubmed research and state of the art about The crosstalk between microRNA and mitochondria in neuro-inflammation. DC and FF coordinators of the paper, participated in drafting the article and in critically revising the article for important intellectual content.

### Conflict of Interest Statement

The authors declare that the research was conducted in the absence of any commercial or financial relationships that could be construed as a potential conflict of interest.
